# Cultural influences on the effects of social norm appeals

**DOI:** 10.1098/rstb.2023.0036

**Published:** 2024-03-11

**Authors:** Rain Wuyu Liu, Maria Knight Lapinski

**Affiliations:** ^1^ Department of Communication, The University of Arizona, Tucson, AZ 85721, Arizona, USA; ^2^ Department of Communication, Michigan State University, East Lansing, MI 48824, USA

**Keywords:** descriptive and injunctive norms, norm message appeals, experiment, food waste prevention, group orientation

## Abstract

This study reports on an experimental test of the effects of descriptive and injunctive norm appeals on intentions to prevent food waste in China and the United States (*N* = 1449), testing the role of cultural context and group orientation in this process. Results showed that the main effects of descriptive and injunctive norm messages on behavioural intentions were mediated by normative perceptions, and cultural context moderated both paths of this mediation. Specifically, with the same message exposure, Chinese participants perceived food waste prevention as more prevalent and socially approved compared to US participants. Normative perceptions interacted with cultural context to influence behavioural intentions, such that both descriptive and injunctive norm perceptions predicted stronger intentions to prevent food waste among Chinese participants compared to Americans. Group orientation yielded a main effect on behavioural intentions, instead of the moderation effects as expected. Findings suggest the need for culturally grounded and contextualized approaches to communication of social norms, as well as building cultural concepts into theories of social norms.

This article is part of the theme issue ‘Social norm change: drivers and consequences’.

## 1. Introduction

In recent decades, the study of social norms has gained popularity across fields including cognitive science, communication and psychology [1–3], investigating normative influence from a variety of theoretical perspectives. Despite the proliferation of scholarship in this area, studies of the role of cultural dynamics in the causal processes associated with normative effects on behaviours remain relatively underappreciated due to a dearth of cross-cultural experimental studies on this issue [4]. Evidence is accumulating for culturally-based differences in the relationship between social norms and behaviours [5,6]. Experiments testing the effects of social norm appeals across multiple cultures/countries are infrequently conducted; hence, questions remain about whether the predictions of social norm theories are replicable in various cultures outside of the Western sphere, where many existing theories were developed and tested [1]. Understanding the effects of culturally-based differences in the strength of social norm appeals on behaviours has theoretical and practical implications for advancing our understanding of the nature of normative influence across different cultures.

**Figure 1.**
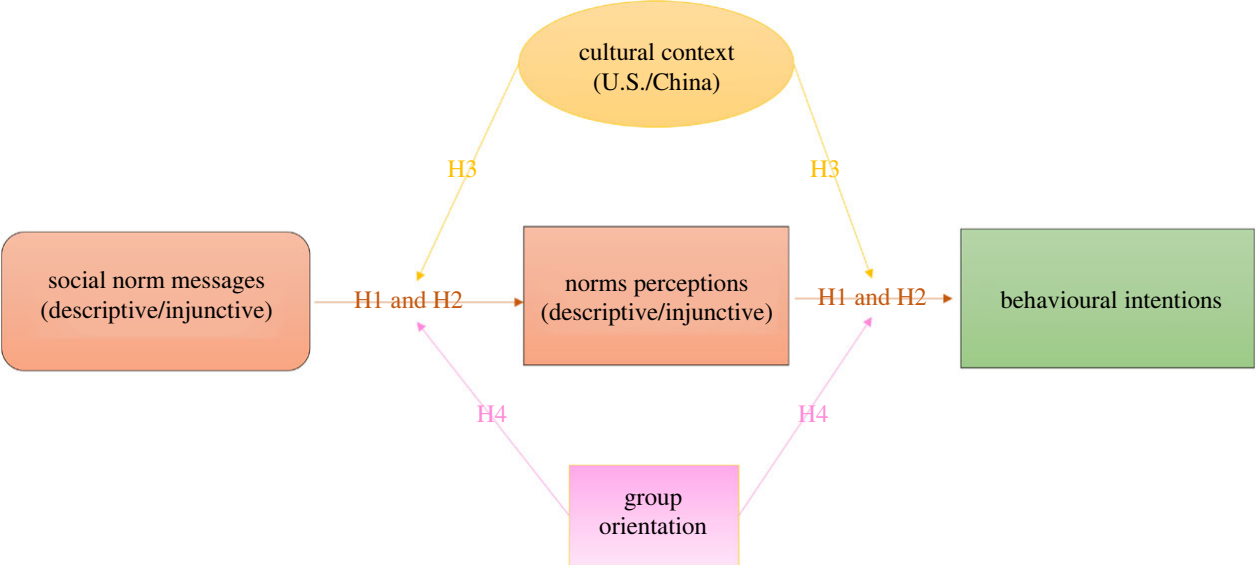
A conceptual framework of the current study illustrating the hypotheses proposed.

To fill this gap, this study investigates the role of cross-cultural differences in normative influence on behavioural outcomes. Specifically, an experiment is conducted to test the effects of descriptive and injunctive norm message stimuli on promoting intentions to reduce food waste, with samples from both China and the US, and to examine the roles of cultural context and group orientation—a form of cultural collectivism—in this process. The study contributes to the social norm literature by expanding the limited cross-cultural evidence that elucidates the mechanisms explaining the effects of social norm messaging in shaping norm perceptions and driving subsequent behaviours. Additionally, these findings can inform the development of culturally grounded communication strategies for promoting conservation behaviours effectively. Figure 1 depicts the conceptual framework of this study, elaborated below.

### (a) Social normative influence on conservation behaviours

Social norms comprise social information indicating what is commonly done and what is commonly approved [7]; normative information is exchanged among group members through social interaction. Cialdini *et al*. [8] conceptualized social norms into two types: *descriptive norms* reflect one's perceptions of the prevalence of a behaviour within a social group (i.e. what is commonly done by others), and *injunctive norms* pertain to one's perceptions of what important others consider as appropriate behaviours (i.e. what is socially approved). The two norms are closely related but conceptually distinct, and they function via separate sources of human motivation [8,9].

#### (i) Descriptive versus injunctive norms

Descriptive norms serve as a decision-making heuristic, offering insight into what actions are prevalent. When substantial ambiguity and novelty are involved in a situation, people tend to rely on these norms to efficiently guide their behaviours, saving time and cognitive effort [9]. Injunctive norms, on the other hand, address the motivation of gaining social approval to build and maintain stable social relations [10]. By specifying what is appropriate and accepted, injunctive norms convey the standards and rules of a group. Individuals are driven to enact a certain behaviour by the potential social rewards or punishments associated with this action [8]. Both types of social norms are well suited to tackle collective action problems, effectively promoting environmental behaviours [11] such as recycling [12], reducing public littering [8], energy conservation [13], choosing alternate fuel vehicles [14] and taking climate actions [15].

When deciding to undertake an action, people may prioritize options that maximize personal benefits [16,17]. When actions are perceived as rare—that is, when the prevalence of the behaviour is low—people may hesitate to act, fearing a loss of personal interest. Conversely, if the behaviour is seen as prevalent, people are more likely to act because the expected benefits of cooperation outweigh individual gains. In this way, descriptive norms offer simple instructions on effective behaviours for achieving common goals, without requiring a complete understanding of complex collective action problems [18].

Meanwhile, people do not make decisions in isolation—social relations and the influence of others matter [19]. Effective injunctive norms inhibit undesirable behaviours through potential social sanctions while offering support and rewards for desirable behaviours, ultimately promoting actions in the collective interest. In situations requiring collective contributions (e.g. engaging in pro-environmental behaviours), concerns over social sanctions, such as ostracism and peer punishment [20] can curb immediate self-interest, addressing public goods problems. Through these social mechanisms, people are encouraged to align their actions with what is prevalent and expected in the group and to prioritize the common good over personal gain, consequently fostering cooperation and collective problem-solving, such as reducing food waste.

#### (ii) The issue of food waste

Food waste has profound impacts on our environment, society and economy. Globally, one-third of all food production is lost or wasted yearly, totaling 1.3 billion tonnes (metric tons) at approximately 1 trillion US$ [21]. Remarkably, just 25% of the wasted food annually could alleviate the hunger of the 690 million people worldwide [21]. Food waste also holds severe environmental consequences, squandering water, land and energy resources. Most food waste ends up in landfills, emitting methane, a greenhouse gas over 25 times more potent than carbon dioxide in trapping heat, and thus contributing to global warming. In fact, food waste generates 8–10% of global greenhouse gas emissions, ranking it as the third-largest contributor, after the US and China, if it were a separate country [22].

This study focuses on the top two food waste-producing countries, China and the US China was ranked at the top of the UNEP Food Waste Index Report 2021, with 90% of Chinese families wasting around 10% of their total food per household [23], around 64 kg food waste per capita per year. Besides, around 350 million tonnes (about 27%) of farm products are ruined and disposed of before reaching retailers, and approximately 45 million tonnes of food are wasted yearly at food stands and restaurants [24]. Likewise, in the US, around 133 billion pounds (≈60.33 million tonnes) of food are discarded annually, representing 30–40% of the food supply, worth more than $161 billion [25]. American consumers throw away 43% of all the food purchased (59 kg food waste per capita per year), which is more than all the retailers combined, leading to an average annual household loss of $1,300 [26]. Educational institutions, including college campuses, significantly contribute to this problem. American college campuses waste around 22 million pounds of food yearly, averaging 142 pounds per student [27], while Chinese university canteens generate about 1.55 million tons of food waste annually, equivalent to 61.03 grams per student per meal [28]. In the light of the global significance of this issue and its specific relevance to the two countries under study, we selected the prevention of food waste among college students as the central focus of this research.

A growing body of literature shows the effects of social norms on preventing or reducing food waste [29]. For instance, in a field experiment in Switzerland, one study [30] found that place cards with informational and normative prompts (combining both descriptive and injunctive norms) helped to reduce food waste in a restaurant by encouraging diners to take away their leftovers. A similar finding on the effects of social norms was also shown in reducing food waste in cross-sectional surveys conducted in Pakistan [31] and Taiwan [32]. Moreover, in another study, experiments involving 3329 citizens found that messages highlighting national or international social norms favouring food waste reduction could boost public support for more ambitious waste reduction goals [33]. Nonetheless, to our best knowledge, there has been little experimental research testing the effects of social norms appeals targeting this critical issue cross-culturally and testing the moderators of their effects. As such, our research is designed to bridge this gap and facilitate our understanding of the solutions to address this global challenge.

In this study, we focus on the impact of norm-based communication, particularly the descriptive and injunctive norms message appeals, on promoting food waste prevention among Chinese and American participants. Meta-analysis [6] has shown that social norms appeals can be effective at shaping normative perceptions, which may subsequently impact behaviours. In line with this, we expect that the message appeals, containing norm information related to food waste prevention, will make participants' norm perceptions salient and hence central to their decisions to act.

Specifically, when behavioural prevalence information is provided through communication, it is expected that a descriptive norm will be accessible to the participants, shaping their perceived descriptive norms (PDN), which serve as social proof to guide the decision-making process. When information concerning the appropriateness and social approval of that action is made salient, perceived injunctive norms (PIN) are expected to be internalized and/or activated to promote norm-consistent behaviours and discourage deviance [34], as individuals aim for social acceptance and relationships while avoiding potential sanctions. Thus, the following hypotheses are posed:

H1: PDN will mediate the effects of descriptive norms messages on behavioural intentions such that relative to those who receive a low-prevalence message, participants receiving a high-prevalence message will perceive a higher prevalence of the behaviour, which will in turn lead to stronger intentions to enact that behaviour.
H2: PIN will mediate the effects of injunctive norms messages on behavioural intentions such that relative to those who receive a weak social approval message, participants receiving a strong social approval message will perceive a stronger approval of the behaviour, which will in turn lead to stronger intentions to enact that behaviour.

### (b) Social norm appeals in cross-cultural context

Susceptibility to social normative influence varies across cultural contexts. There is evidence for culturally-based differences in the effectiveness of norm appeals on behaviours [6] and the cultural variations in the strengths of social norms on behavioural compliance [35]. Here, we focus on testing the role of cultural dynamics in the process of shaping norm perceptions following exposure to norm messages, as well as the effects of normative perceptions on focal behavioural outcomes.

#### (i) Cultural context and normative influence

Norm perceptions are subjective and dynamic, developed via three primary sources [36], including (1) observable behaviours of others, especially among one's important social groups, termed referent groups, (2) direct and indirect communication, including both explicit and implicit words and deeds, and (3) knowledge of the self (i.e. one's own preferences and attitudes, and the belief that most others think and behave in the same manner as oneself).

In addition to these sources, research underscores the important role of institutional signals in shaping norm perceptions [37,38], such as governments, schools and the mass media [39]. These social institutions can shape perceptions of common or desirable behaviour within a group by directly communicating norms through institutional signals/messages, or indirectly, when people observe behavioural changes due to institutional actions and adjust their perceptions of the norms [38].

In a comprehensive review of the history of China's food waste policies since 2012 and the recent adoption of the anti-food waste law in 2021, Feng *et al*. [40] delve into the reasons why the Chinese government chose to implement a law with sanctions to combat food waste, targeting the catering industry. It suggests that cultural aspects, particularly traditional Chinese dining habits and pop culture, are challenging efforts to reduce food waste without legal sanctions. Though households are not the focus of the current law, given that the booming catering industry generates more waste than households and is easier to monitor and regulate, the Chinese central government has been introducing anti-food waste policies for a long time, dating back to 1951 when Mao Zedong initiated a campaign against the ‘three evils': corruption, waste and bureaucracy ([40], p. 463).

In the US, the Department of Agriculture (USDA) and Environmental Protection Agency (EPA) [41] announced the first-ever domestic goal to reduce food loss and waste on 16 September 2015, aiming to cut food loss and waste in half by 2030. To achieve this goal, the EPA has laid out plans to work with various stakeholders in the food system, including the private sector, government, non-profit organizations, academia and faith-based groups. The emphasis is on a holistic approach to encourage everyone to take action to minimize food waste from the very beginning, retain valuable food resources and manage food waste sustainably. Despite these efforts, the EPA's latest progress update in April 2023 reveals a significant challenge in achieving the 2030 food waste reduction goal. Between 2018 and 2019, food waste per person increased from 335 to 349 pounds, and over the three-year period from 2016 to 2019, there was a six percent *per capita* food waste increase [41].

When comparing the institutional signals related to the focal issue from the governmental agencies of these two countries, it is likely that Chinese participants, when exposed to the same norm message, may perceive stronger norms regarding both the prevalence and social approval of preventing food waste in contrast to their American counterparts. This perception is not solely influenced by the social norm information conveyed in the message; participants may also draw upon their beliefs and real-life observations of norms associated with the targeted behaviour within their cultural context. Considering potential variations in the actual prevalence and social approval of food waste prevention within the social circles of individuals, it is conceivable that perceived norms might differ between the two national samples.

Goyal *et al*. [42] conducted experiments involving American and Indian participants to explore how culture shapes norm perceptions and categorizations. Findings revealed that when different cultures possess distinct norms for the same situation, such as reciprocity and spiritual purity norms in their study, culture influences not only the significance that individuals attribute to information but also the interpretation of the information itself. This cultural influence, in turn, affects memory by shaping categorization, determining whether a behaviour is regarded as norm-consistent or norm-violating. Therefore, the salience of the same norm messages may be interpreted and internalized differently, resulting in a different level of norm perceptions cross-culturally.

Furthermore, cultural context can exert a substantial influence on the impact of norms on behavioural outcomes. One specific construct associated with the adherence to social norms and the punishment of norm violators is *cultural tightness and looseness* (CTL; [35,43]). The key components of CTL address the strength of social norms (i.e. how clear and pervasive norms are within societies) and the strength of sanctioning (i.e. how much tolerance there is for deviance from norms within societies).

Existing literature on CTL [43–45] indicates that cultural groups that have had high degrees of territorial threats necessitating national defense, low levels of natural resources (e.g. food supply relative to population size) and high degrees of natural disasters (e.g. floods, cyclones and droughts)—such as China (with a tightness score of 7.9)—evolve to be tight, that is, have stronger norms and less tolerance for deviant behaviour, in order to coordinate social action. On the other hand, cultural groups that generally have low threats, an abundance of natural resources and relatively fewer natural disasters—such as the US (with a tightness score of 5.1)—evolve to be loose, i.e. have weaker norms and more tolerance for deviant behaviour. As such, we anticipate that Chinese participants will be more susceptible to the normative effects of adopting the perceived prevalent and socially endorsed behaviour, as evidenced in this recent meta-analysis [46]. Taken together, the following hypothesis is proposed:

H3: Cultural context (i.e. country of origin) will moderate how norms messages shape normative perceptions and the link between normative perceptions and behaviours such that relative to US participants, Chinese participants' perceptions will be more influenced by norm messages, which will in turn, lead to stronger intentions to enact the behaviour.

#### (ii) Group orientations and normative influence

Norm sensitivity is often discussed as a function of differences in group or collective orientation (i.e. the extent to which an individual is willing to prioritize group goals over personal goals), and cultures shape our views toward the individual–group relationship. Individualism–collectivism [47] operates at the *societal or cultural level*, distinguished based on the degree to which members of the society relate to a collective, usually a stable ingroup (e.g. family, nation or tribe). In collectivistic cultures, individuals prioritize group harmony and conformity, adhering to social expectations and norms to maintain social cohesion. Social norms hold substantial influence in these cultures, as individuals prioritize collective well-being to avoid standing out. Conversely, in individualistic cultures, individuals prioritize personal goals and preferences over social expectations, emphasizing personal autonomy and independence. While social norms still play a role in individualistic cultures, greater individual variation and deviation from norms are permitted. The meta-analysis conducted by Fischer & Karl [46] showed that collectivism (versus individualism) strengthened normative effects on compliance with COVID-19-related behaviours at the country level.

Group or collectivistic orientation captures the individualism–collectivism dimension at the *individual level*, referring to one's inclination toward collective or group-oriented behaviour within their personal mindset or behaviour patterns [48]. Group orientation is culturally-based, as individuals in collectivistic cultures are more likely to exhibit a group-oriented mindset, whereas those from individualistic cultures may exhibit varying degrees of group orientation depending on their personal inclinations and experiences [49]. Nonetheless, it is crucial to acknowledge that the literature on individualism–collectivism has faced criticism for making broad generalizations about individuals from diverse cultures [50]. In this study, we aimed to address this issue by measuring group orientation at the individual level in both national samples, allowing for a more nuanced understanding of individuals' tendencies toward collective cooperation while controlling for the effects of their country of origin.

Compared to individual-oriented people, who tend to pursue their personal interests and emphasize uniqueness, group-oriented individuals tend to prioritize maintaining group harmony, adhering to social norms and fulfilling collective responsibilities. They may experience positive affect when behaving in accordance with norms [51] and recognize that their compliance is supported by others in the group. Hence, group-oriented individuals are more likely to be influenced by social norms to shape their perceptions and adopt or adjust their behaviours, whereas individual-oriented ones are more likely to be guided by their own beliefs, values and attitudes when making behavioural decisions [52].

Lapinski *et al*. [53] examined how group orientation interacted with descriptive norm messages in promoting water conservation behaviours in the US. They found that group orientation moderated the impact of descriptive norms on attitudes and behavioural intent, such that, among individuals with strong group orientation, descriptive norms had no influence on their attitudes or behaviours; they maintained positive attitudes and intentions regardless of the prevalence information. Conversely, among those with a weak group orientation (more individualistic), descriptive norms were negatively associated with their attitudes and intentions. They suggested that individualistically oriented individuals with the perception that most others engage in a behaviour held more negative attitudes and weaker intentions, possibly identifying with the minority who do not engage in that behaviour. Sherman *et al*. [54] studied how collectivistic orientation (measured at the individual level) and socioeconomic status (SES) interacted to influence environmental support, particularly in the context of climate change, with a US sample. Results showed that descriptive norms had the strongest impact on environmental support for individuals high in both collectivism and SES. Notably, both the Lapinski *et al*. [53] and the Sherman *et al*. [54] studies were limited by samples in the US, which may have restricted the range of group orientation.

In a cross-cultural study involving American and Chinese participants, Saracevic *et al*. [55] tested both descriptive and injunctive norm appeals on pro-environmental behavioural intentions. They manipulated the level of self-construal (individual-level cultural orientation: independent or interdependent) among all participants with experiment message stimuli. They found that although both norm appeals had a stronger effect among Chinese participants in general, the influence of these appeals changes depending on whether independent or interdependent self-construal was activated. Based on the existing evidence indicating the impact of culturally-based group orientations on people's susceptibility to normative influence, the following hypothesis is proposed:

H4: Group orientation will moderate the effects of norms messages on normative perceptions and the link with behavioural intentions, such that compared to those who are more individual-oriented, group-oriented participants' normative perceptions will be more influenced by norm messages, which will in turn, lead to stronger intentions to enact the behaviour.

## 2. Method

A 2 (descriptive norms: high versus low) × 2 (injunctive norms: strong versus weak) between-subject experiment was conducted among samples from the US and China to test hypotheses and answer research questions. Participants were randomly assigned to one of the four experimental conditions. Prior to the data collection, G*Power software was used to determine an appropriate sample size [56], considering the desired level of significance (*α* = 0.05) and statistical power (0.90), the analyses to be conducted (ANOVA and regression), the estimated number of predictors in the statistical models, the estimated effect size (0.23 to 0.17, based on the recent meta-analysis; [6]) and the potential attrition rate (US–20% and China–50%). Considering these criteria, we aimed for a total estimated sample size of 1263, with 560 from the U.S. and 700 from China. All study procedures were reviewed and approved by an institutional review board (IRB) in a U.S. university^1^.

### (a) Participants

In total, 1449 participants were recruited from the U.S. (*n* = 563) and mainland China (*n* = 886), respectively. The U.S. participants (U.S. citizens or permanent residents only) were recruited from the research participant pool in a large Midwestern university, and Chinese participants (Chinese citizens only) were recruited among students in three major universities in Yunnan Province^2^. Participants from both countries were incentivized with course credit.

The U.S. sample was predominantly female (66.1%), with a mean age of 19.79 (18 to 41, *s.d.* = 1.79). The sample was relatively evenly distributed across years in school. Most participants reported being Caucasian (82.1%), with 7.8% African American, 3.7% Asian, and the remaining comprising other groups. The Chinese sample was predominantly female (66.6%), with a mean age of 20.45 (18 to 28, *s.d.* = 1.89). Most participants reported being Han Chinese (87.9%); other ethnic groups represented included Bai, Yi and Hui Chinese ethnic minorities.

### (b) Procedure

The research team included researchers from China and the U.S. who collaborated on the design and implementation of all study materials; every effort was made to ensure conceptual equivalence of study materials while addressing key, culture-bound issues associated with the study topic. American participants completed study materials in English, and Chinese participants completed them in Mandarin Chinese. A professional English–Chinese translator who was not involved in the research and blind to the study predictions first translated the original English experimental materials and instruments into Chinese. Then, another professional English–Chinese translator back-translated the study materials from Chinese into English. Both English and Chinese versions of the questionnaires were carefully checked and compared by the research team [57] and edited to maximize conceptual equivalence.

In the US, data were collected online with Qualtrics (see https://www.qualtrics.com). In China, paper-based surveys were used, according to the request of the collaborators who assisted in the data collection process^3^. Group orientation was measured first. Next, participants were informed that they were being asked to take part in a study in collaboration with the Culinary Services and Campus Sustainability at their university to help launch an on-campus sustainability programme. Participants were guided to read a descriptive paragraph (described below) and were randomly assigned to one of the four experimental conditions. Immediately following the message, participants completed measures of perceived descriptive norms and perceived social approval of preventing food waste, behavioural intent to reduce food waste in the future, perceived believability of the message and demographics. Upon completion of the study, participants received a thorough debriefing, including an explanation of the study's purpose, the use of deception in the messages and the researchers’ contact information for follow-up questions or concerns. Data were captured automatically as American participants completed the study. Researchers fluent in both English and Chinese entered the data for the Chinese sample. Cross-checks were performed periodically to ensure the accuracy of the data entered into the computer, and data were carefully cleaned once data entry was completed.

### (c) Message stimuli^4^

Both injunctive and descriptive norm messages were developed based on state-of-the-art recommendations for the social norms message design [58] and shown to the participants simultaneously. Norm messages were embedded in a passage using the cover story of launching an on-campus food waste prevention programme, *Waste Wise*. The passages across conditions were identical in content, length, and the nature of the arguments presented. The only variation was the descriptive and injunctive norm manipulations. Participants were informed that to launch this program successfully, a large-scale survey had been conducted among students in their university, and the data presented in the messages were from the results of that study.

#### (i) Descriptive norms

For the descriptive norm manipulations, the messages varied in the reported prevalence of the behaviour. In the *high-prevalence condition*, the message said that ‘the results showed that most of the students at XX University, about 80%, have taken actions to help reduce food waste.’ In the *low-prevalence condition*, the message said that ‘the results showed that only a few of students at XX University, about 20%, have taken actions to help reduce food waste.’

#### (ii) Injunctive norms

For the injunctive norm manipulations, the messages varied regarding the degree of social approval toward the behaviour. In the *strong social approval condition*, the message said that ‘the majority of students have indicated that they believe preventing food waste is very important and that wasting food is definitely an unacceptable and despicable behaviour for XX University students.’ In the *weak social approval condition*, the message said that ‘only a small number of students have indicated that they believe preventing food waste is very important and wasting is definitely an unacceptable and despicable behaviour for XX University students.’

### (d) Measurement

Scale items used in this study were adapted from previous research to be appropriate for the topic and cultural context. Except for those noted, items were measured on 5-point Likert scales ranging from 1 (strongly disagree) to 5 (strongly agree). Reliability estimates, zero-order correlations, means and standard deviations of all measures for each national sample are reported in table 1.

**Table 1.** Zero-order correlations, means and standard deviations of measured variables in the U.S. American and Chinese Samples. PDN, perceived descriptive norms; PIN, perceived injunctive norms; IND, individual-oriented; COL, collective-oriented; GO, group orientation (GO is computed by subtracting IND from COL; see §2c(i)), BI, behavioural intentions; ATT, attitudes; BEL, message believability; *m*, mean; *sd*, standard deviation.

	US (*N* = 563)								China (*N* = 886)							
	PDN	PIN	IND	COL	GO	BI	ATT	BEL	PDN	PIN	IND	COL	GO	BI	ATT	BEL
PDN	**0.78**								**0.74**							
PIN	0.42**	**0.81**							0.22**	**0.83**						
IND	−0.01	0.03	**0.70**						0.04	0.11**	**0.73**					
COL	0.09*	0.13**	−0.07	**0.77**					0.06	0.23**	0.17**	**0.77**				
GO	0.07	0.06	−0.72**	0.74**	—				0.01	0.06	−0.75**	0.52**	—			
BI	0.36**	0.44**	−0.07	0.64**	0.49**	**0.87**			0.52**	0.50**	0.02	0.40**	0.25**	**0.88**		
ATT	−0.05	0.17**	0.14**	0.16**	0.02	0.22**	**0.82**		0.05	0.23**	0.15**	0.30**	0.08*	0.23**	**0.80**	
BEL	−0.08	0.10*	0.03	0.08	0.03	0.14**	0.43**	**0.89**	0.13**	0.10**	0.01	0.19**	0.12**	0.20**	0.22**	**0.93**
*m*	2.78	2.65	3.82	3.64	−0.18	2.98	4.22	4.10	3.36	3.66	3.27	3.55	0.28	4.10	4.42	3.97
*sd*	0.70	0.58	0.41	0.42	0.61	0.78	0.59	0.64	0.63	0.50	0.46	0.36	0.54	0.52	0.46	0.81

*Note:* Scale reliabilities are presented on the diagonal in bold. **Correlation is significant at the 0.01 level (2-tailed). *Correlation is significant at the 0.05 level (2-tailed).

#### (i) Group orientation

The extent to which an individual is oriented toward an in-group in their culture was measured by a 9-item scale derived from Triandis' [48] individualism–collectivism scale. It consists of 4 items measuring one's tendency to set priority on personal goals over group goals (individual-oriented; e.g. ‘I enjoy being unique and different from others in many ways.’), with the other 5 items measuring one's tendency to set priority on group goals over personal goals (collective-oriented; e.g. ‘It is important for me to maintain harmony within my group’). Group orientation was computed by subtracting the mean score of individual-oriented items from the mean score of collective-oriented items [59]. Higher scores indicated a stronger orientation toward the group.

#### (ii) Attitudes

Participants’ attitudes toward food waste prevention were measured with four items adapted from Lapinski *et al*. [53]. Higher scores indicated a more favourable attitude towards food waste prevention. Sample items include ‘I think reducing food waste is a good idea’ and ‘I feel strongly preventing food waste is important.’

#### (iii) Perceived descriptive norm

PDN were assessed with five items adapted from Park & Smith [60], with higher scores indicating a greater perception of prevalence. Sample items include ‘I believe most students in my school engage in behaviours to help reduce food waste.’

#### (iv) Perceived injunctive norm

PIN were assessed with four items developed by Park and Smith [60]. Higher scores indicated a perception of a greater injunctive norm. Sample items include ‘It is clear that the majority of xx university students around me support taking actions to reduce food waste.’

#### (v) Behavioural intent

Participants’ post-experiment intent to engage in future food waste prevention activities was measured with six items adapted from Park and Smith [60]. Higher scores indicated a stronger intention to engage in future food waste prevention activities. Sample items include ‘I have it in my mind to start meal planning to reduce unnecessary food waste.’

#### (vi) Message believability

To check that participants were not suspicious of the background story, the believability of the message was assessed using seven 5-point semantic differential items derived from Beltramini's [61] perceived believability scale. Higher scores indicated greater believability of the message. Sample anchors include ‘believable/unbelievable’ and ‘credible/not credible.’

#### (vii) Demographics

Standard demographic information was collected at the end of the experiment, including gender, age, major, year in school, ethnicity, how many times in a typical week that participant ate in the dining halls on campus, and how much money participants had available to spend every month on food.

## 3. Results

### (a) Measurement invariance and preliminary analysis

Multi-group confirmatory factor analysis (MGCFA) was conducted for all measures using *Mplus* [62], following the procedures suggested by Byrne [63].^5^ The purpose was to establish unidimensional measurement models prior to computing composite scores and testing hypotheses, and to provide evidence that the observed scale indicators/items under study measured the same theoretical constructs (latent variables or factors) across the two national samples. Missing data were handled with listwise deletion. The results (see electronic supplementary material, appendix B) showed that except for the scale of collective-orientated, no significant changes occurred to chi-squares across the three models, indicating a good measurement equivalence across the two samples. For the collective-orientated scale, although the initial model of scalar invariance was rejected, the value scale met the partial scalar invariance test [64]. Hence, the results were deemed acceptable for additional analysis.

Data were first analysed with hierarchical linear modelling [65] since individuals were nested in their respective countries. The multilevel analysis allowed for the partitioning of variance in the outcome variable attributed to both individual-level and country-level differences. The intraclass correlation coefficient on behavioural intentions was 0.03, and the variance in behavioural intentions across countries was not significantly different from zero, *p* = 0.96, indicating that there was minimal or no clustering of the outcome variable at the country level. Hence, the subsequent analysis was conducted at the mono-level.

We also accessed the group orientation across the two samples with an independent-samples *t*-test. The results showed that the Chinese sample (*M* = 0.28, *s.d.* = 0.54) was significantly more group-oriented compared to those in the U.S. sample (*M* = −0.18, *s.d.* = 0.61), *t*(1435) = 15.34, *p* < 0.001, Cohen's *d* = 0.83, suggesting that this individual-level construct was influenced and reflecting the individualism–collectivism dimension at the country-level [47].

### (b) Manipulation checks

Analysis of covariance (ANCOVA) was conducted using the whole sample to check if the message stimuli induced the specific type of norms as expected, controlling for cultural context (country of origin), attitudes and demographic variables. Descriptive and injunctive norm stimuli were treated as the independent group factors, with either PDN or PIN as the dependent variable. Detailed results are reported in the supplementary file (electronic supplementary material, appendix C).

Overall, there was a main effect of the descriptive norm message stimuli on PDN, such that participants in the high-prevalence condition rated food waste prevention behaviour as significantly more prevalent than those in the low-prevalence condition. There was also a main effect of the injunctive norm message stimuli on PIN, such that participants in the strong social approval condition rated food waste prevention behaviour as significantly more socially approved than those in the weak social approval condition. No statistically significant interaction between descriptive and injunctive norms stimuli was found to influence PDN or PIN. Hence, the message stimuli were deemed successful.

Following prior research on social norms appeals [66], the perceived believability of the message stimuli was assessed with one-sample *t*-tests and ANCOVA in the whole sample, with message conditions and cultural context as the fixed factors, controlling for the same aforementioned set of demographics variables. The ANCOVA results (see electronic supplementary material, appendix C) indicated that messages were perceived as similarly believable across all experimental conditions, *F*_messages_ (3, 1335) = 0.26, *p* = 0.85, and two cultures, *F*_country_ (1, 1335) = 0.74, *p* = 0.57. One sample *t*-tests showed that the mean believability score in each experimental condition in both national samples was significantly higher than the midpoint of the scale, ranging from 3.95 to 4.12, all *p* < 0.001. Hence, all message stimuli were deemed similarly believable.

### (c) Hypotheses testing

To gain a holistic view of the underlying relationships among the key variables proposed in the hypotheses, two moderated mediation models were tested using the PROCESS Macro Model 75 [67]; see the conceptual diagram in electronic supplementary material, appendix D), with either type of norm message (descriptive or injunctive norm appeal) as the predictor, the associated norm perceptions as the mediator (PDN or PIN), both cultural context (i.e. country of origin; dummy variable, US = 0; China = 1) and group orientation as the moderators, and behavioural intentions as the outcome variable, controlling for participants' age, sex, year at school, frequency of eating at the dining hall, message believability and attitudes toward the focal issue. Continuous variables (PDN, PIN and group orientations) were mean-centred in testing the interaction effects to avoid multicollinearity and enhance the interpretation of regression coefficients. Robustness checks were conducted to examine model stability and control for potential confounding variables (see electronic supplementary material, appendix E).

**Figure 2.**
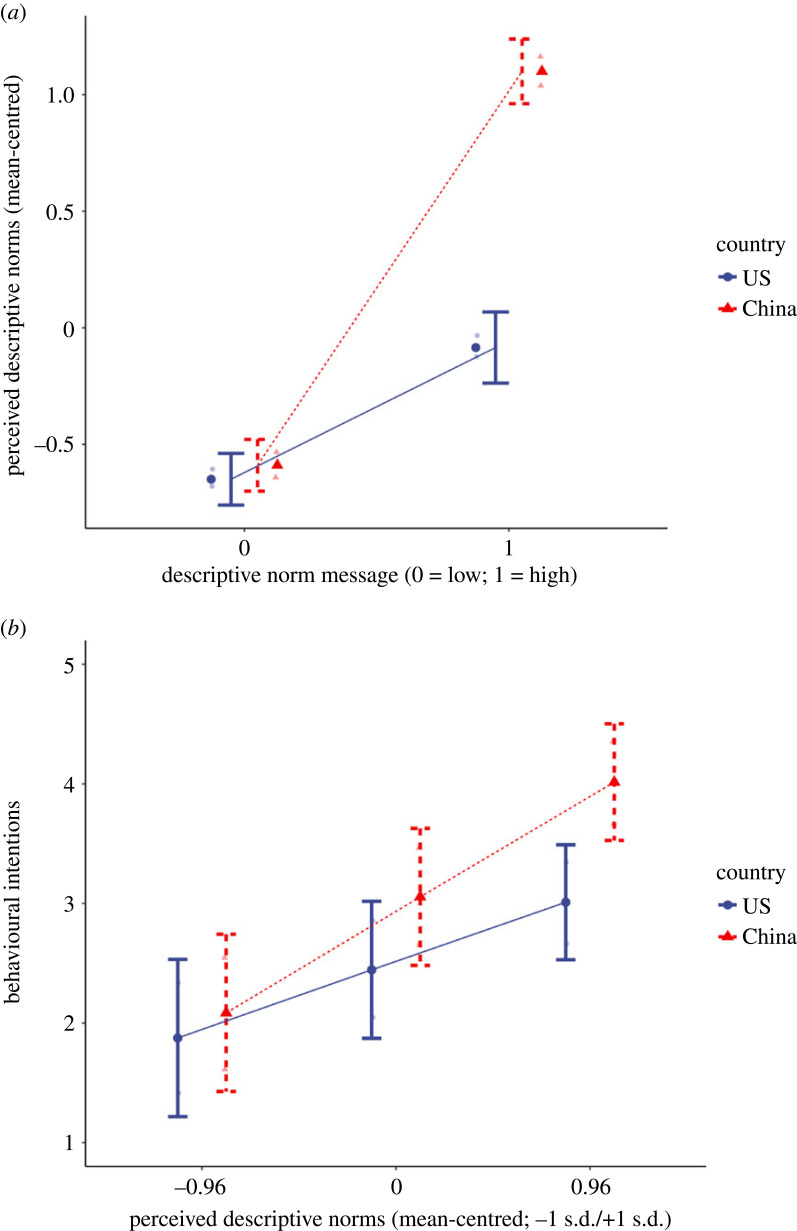
Plots of interactions between (*a*) descriptive norm message and cultural context on perceived descriptive norms (top) and (*b*) perceived descriptive norms and cultural context on behavioural intentions (bottom).

H1 (PDN as the mediator between descriptive norm message and intentions), H3 (cultural context moderates the mediational path in H1) and H4 (group orientation moderates the mediational path in H1) were tested in the descriptive norm appeal model. The results (table 2) showed that the overall model, including all the predictors and covariates, was significant, *F*_12,1273_ = 124.90, *p* < 0.001, adjusted *R*^2^ = 0.54. Consistent with the manipulation check, the descriptive norms message yielded a significant main effect on PDN, controlling for the cultural context (i.e. country of origin) and group orientation, neither of which yielded a main effect. As predicted in H3, the descriptive norms message interacted with the cultural context in predicting PDN (see the top panel in figure 2 for the plot of the interaction), such that with the same message exposure, Chinese participants (conditional effect of the message on PDN = 1.69, *s.e.* = 0.05, *t* = 36.03, *p* < 0.001) perceived a more prevalent norm of preventing food waste among peers of their university (i.e. the referent group) compared to Americans (conditional effect of the message on PDN = 0.57, *s.e.* = 0.05, *t* = 10.49, *p* < 0.001). However, no significant interaction was found between the message and group orientation in predicting PDN, which is inconsistent with H4.

**Table 2.** Effects of descriptive norm message on behavioural intentions mediated by perceived descriptive norms (H1), and moderated by cultural context (H3) and group orientations (H4)*.* PDN, perceived descriptive norms; GO, group orientation; BI, behavioural intentions; LLCI, lower limit confidence interval; ULCI, upper limit confidence interval.

*N* = 1286	path coefficient (*b*)	*se* *b*	*t*	*p*	LLCI	ULCI	total *R*^2^
covariates							
age	−0.01	0.01	−0.79	0.43	−0.04	0.02	
sex	−0.01	0.01	−1.37	0.17	−0.02	0.00	
year at school	−0.01	0.02	−0.56	0.58	−0.06	0.03	
eat at dinning hall	0.00	0.00	0.06	0.95	−0.01	0.01	
attitudes	−0.01	0.03	−0.28	0.78	−0.08	0.06	
message believability	0.04	0.02	1.69	0.09	−0.01	0.09	
outcome variable: PDN							**0**.49*
constant	−0.51	0.28	−1.84	0.07	−1.06	0.03	
*a*_1_: message (low = 0) → PDN	0.55	0.05	9.75	<0.001	0.44	0.66	
*a*_2_: culture (US = 0) → PDN	0.06	0.06	1.06	0.29	−0.05	0.17	
*a*_3_: GO → PDN	0.06	0.04	1.48	0.14	−0.02	0.15	
*a*_4_: message × culture → PDN	1.13	0.07	15.16	<0.001	0.98	1.27	
*a*_5_: message × GO → PDN	0.03	0.06	0.38	0.71	−0.10	0.14	
outcome variable: BI							**0**.54*
constant	1.34	0.22	5.92	<0.001	0.90	1.79	
*b*_1_: PDN → BI	0.10	0.03	3.06	<0.005	0.45	0.73	
*b*_2_: country → BI	0.48	0.03	7.94	<0.001	0.46	0.76	
*b*_3_: GO → BI	0.66	0.05	12.55	<0.001	0.56	0.77	
*b*_4_: PDN × culture → BI	0.41	0.08	5.16	<0.001	0.26	0.57	
*b*_5_: PDN × GO → BI	−0.10	0.05	−1.95	0.05	−0.21	0.00	
*c*′: message → BI	−0.02	0.03	−0.68	0.50	−0.07	0.04	

*Note*: *p < 0.001. Direct effect of *Message* on *Intentions* = *c*′ (the intercept), not considering mediation or moderation. Conditional indirect effect of *Message* on *Intentions* through the mediator *PDN* = (*a*_1_ + *a*_4_*Culture + a_5_GO*) * (*b*_1_ + *b*_4_*Culture + b*_5_*GO*).

Furthermore, PDN, cultural context and group orientation all yielded a significant main effect in predicting intentions of preventing food waste. PDN interacted with cultural context in predicting behavioural intentions (see the bottom panel in figure 2 for the plot of the interaction), such that Chinese participants are more susceptible to the descriptive normative influence in expressing stronger intentions to prevent food waste (conditional effect of PDN on intentions = 1.00, *se* = 0.05, *t* = 19.69, *p* < 0.001), compared to Americans (conditional effect of the message on PDN = 0.59, *se* = 0.07, *t* = 8.48, *p* < 0.001). No significant interaction was found between PDN and group orientation in predicting behavioural intentions. Overall, the direct effect of the descriptive norm message on behavioural intentions was not significant (direct effect = −0.02, *se* = 0.03, *t* = −0.68, *p* = 0.5), but the conditional indirect effects of descriptive norm message → PDN → behavioural intentions moderated by cultural context was significant, with pairwise contrast between indirect effect among Chinese (indirect effect = 1.70, *BootSE* = 0.09, *BootCI* [1.53, 1.88]) and Americans (indirect effect = 0.33, *BootSE* = 0.06, *BootCI* [0.23, 0.45]) shown significant (*BootCI* [1.17, 1.57]). No significant indirect effect was found moderated by group orientations. Hence, for descriptive norms, tdata were consistent with H1 and H3, but inconsistent with H4.

**Figure 3.**
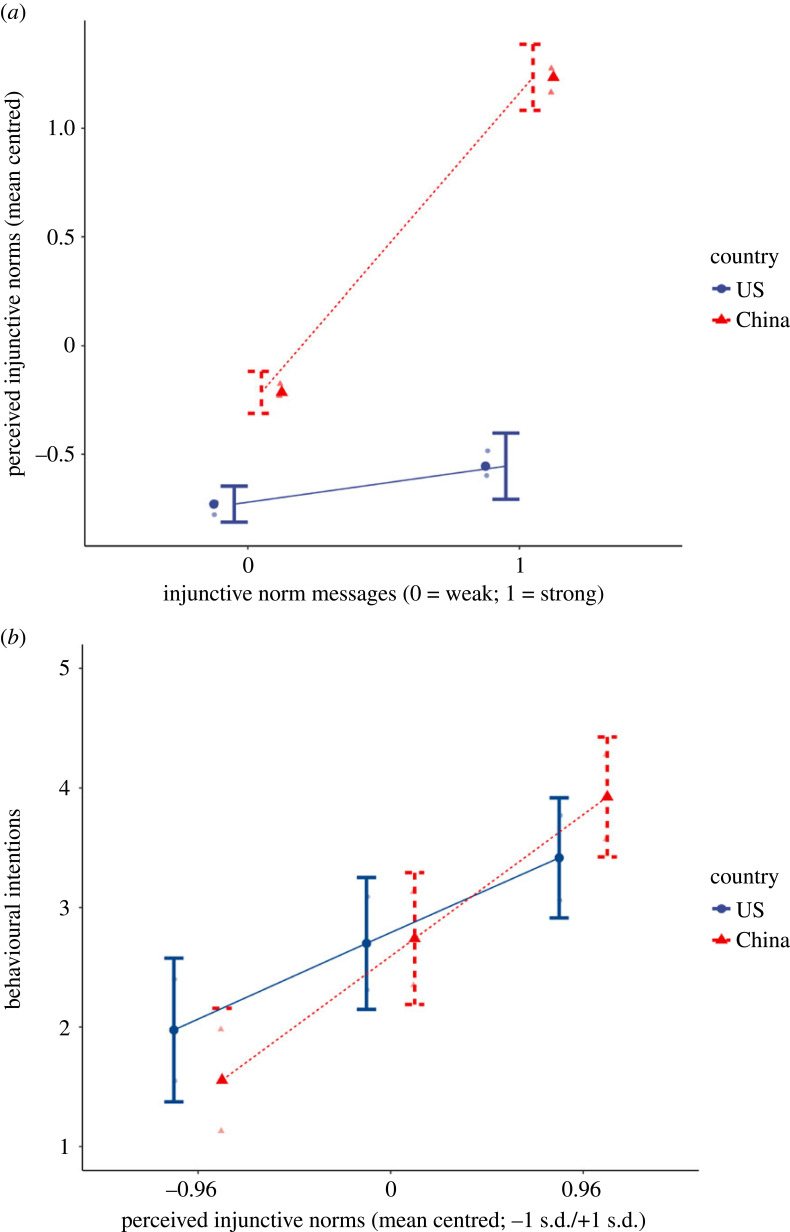
Plots of interactions between injunctive norm message and cultural context on perceived injunctive norms (*a*) and perceived injunctive norms and cultural context on behavioural intentions (*b*).

H2 (perceive injunctive norms (PIN) as the mediator between descriptive norm message and intentions), H3 (cultural context moderates the mediational path in H2) and H4 (group orientation moderates the mediational path in H2) were tested in the injunctive norms appeal model. The results (table 3) showed that the overall model, including all the predictors and covariates, was significant, *F*_12,1278_ = 123.47, *p* < 0.001, adjusted *R*^2^ = 0.53. Consistent with the manipulation check, injunctive norm message yielded a significant main effect on PIN, controlling for the cultural context and group orientation. Meanwhile, cultural context exerted a significant main effect on PIN, such that Chinese participants perceived a stronger social approval in preventing food waste compared to their American counterparts, controlling for all other factors, including the message stimuli. Consistent with H3, the injunctive norm message interacted with the cultural context in predicting PIN (see figure 3*a* for the plot of the interaction), such that with the same message exposure, Chinese participants (conditional effect of the message on PIN = 1.45, *se* = 0.04, *t* = 38.23, *p* < 0.001) perceived a stronger injunctive norm of preventing food waste among peers of their university compared to Americans (conditional effect of the message on PIN = 0.18, *se* = 0.04, *t* = 4.02, *p* < 0.001). However, no significant interaction was found between the message and group orientation in predicting PIN, which is inconsistent with H4.

**Table 3.** Effects of injunctive norm message on behavioural intentions mediated by perceived injunctive norms (H2) and moderated by cultural context (H3) and group orientations (H4). PIN, perceived injunctive norms; GO, group orientation; BI, behavioural intentions; LLCI, lower limit confidence interval; ULCI, upper limit confidence interval.

*N* = 1291	path coefficient (*b*)	*se b*	*t*	*p*	LLCI	ULCI	total *R*^2^
covariates							
age	0.00	0.01	0.19	0.85	−0.02	0.02	
sex	0.00	0.01	−0.58	0.56	−0.01	0.01	
year at School	0.00	0.02	0.24	0.81	−0.03	0.04	
eat at dinning hall	0.00	0.00	0.94	0.35	0.00	0.01	
attitudes	0.20	0.03	7.00	<0.001	0.14	0.25	
message believability	0.06	0.02	2.83	<0.005	0.02	0.09	
outcome variable: PIN							**0**.40*
constant	−1.87	0.22	−8.37	<0.001	−2.31	−1.43	
*a*_1_: message (weak = 0) → PIN	0.18	0.04	4.02	0.00	0.09	0.26	
*a*_2_: culture (US = 0) → PIN	0.52	0.04	11.60	0.00	0.43	0.60	
*a*_3_: GO → PIN	0.05	0.03	1.54	0.12	−0.01	0.12	
*a*_4_: message × culture → PIN	1.27	0.06	21.24	0.00	1.15	1.39	
*a*_5_: message × GO → PIN	0.04	0.05	0.77	0.44	−0.06	0.13	
outcome variable: BI							**0**.53*
constant	2.27	0.49	4.60	<0.001	1.30	3.23	
*b*_1_: PIN → BI	0.27	0.04	5.94	<0.001	0.14	0.49	
*b*_2_: country → BI	0.04	0.10	0.45	0.65	−0.14	0.23	
*b*_3_: GO → BI	0.64	0.05	12.15	<0.001	0.54	0.74	
*b*_4_: PIN × culture → BI	0.48	0.10	4.80	<0.001	0.29	0.68	
*b*_5_: PIN × GO → BI	−0.06	0.05	−1.10	0.27	−0.16	0.04	
*c*′: message → BI	0.03	0.02	0.13	0.9	−0.05	0.05	

*Note*: *p < 0.001. Direct effect of *Message* on *Intentions* = *c*′ (the intercept), not considering mediation or moderation. Conditional indirect effect of *Message* on *Intentions* through the mediator *PIN* = (*a*_1_ + *a*_4_*Culture + a*_5_*GO*) * (*b*_1_ + *b*_4_*Culture + b*_5_*GO*).

Furthermore, PIN and group orientation yielded a significant main effect in predicting intentions of preventing food waste, but no main effect was shown for cultural context. Instead, PIN interacted with cultural context in predicting behavioural intentions (see figure 3*b* for the plot of the interaction), such that Chinese participants are more susceptible to the injunctive normative influence in expressing stronger intentions of preventing food waste (conditional effect of PIN on intentions = 1.23, *se* = 0.06, *t* = 20.26, *p* < 0.001) compared to Americans (conditional effect of the message on PIN = 0.75, *se* = 0.08, *t* = 8.94, *p* < 0.001). No significant interaction was found between PIN and group orientation in predicting behavioural intentions. Overall, the direct effect of injunctive norm message on behavioural intentions was not significant (direct effect = 0.00, *se* = 0.03, *t* = 0.13, *p* = 0.90), but the conditional indirect effects of injunctive norm message → PIN → behavioural intentions moderated by cultural context was significant, with pairwise contrast between indirect effect among Chinese (indirect effect = 1.78, *BootSE* = 0.09, *BootCI* [1.61, 1.95]) and Americans (indirect effect = 0.13, *BootSE* = 0.04, *BootCI* [0.06, 0.22]) also shown to be significant (*BootCI* [1.46, 1.84]). No significant indirect effect was found moderated by group orientations. Hence, data were consistent with H2 and H3 ((for injunctive norms), but inconsistent with H4 (for injunctive norms).

## 4. Discussion

Despite the importance of social norms in influencing human behaviours, questions remain about the causal mechanisms underlying the effects of social norms across diverse cultural groups. By experimentally manipulating the salience of both descriptive and injunctive norm perceptions through persuasive messages and scrutinizing their subsequent effects with samples from China and the U.S., our research not only advances norm scholarship by deepening our knowledge of the cultural dimensions of normative influence, but also holds valuable practical implications. By unveiling the ways in which social norms appeals can be harnessed as potent tools for promoting pro-environmental behaviours, this research offers a promising avenue for encouraging positive change.

Consistent with previous literature on the effects of social norms appeals in the U.S. [6], we successfully replicated a well-established mediation model, reaffirming the role of norm messages in shaping normative perceptions and, consequently, influencing behavioural intentions. This not only underscores the robustness of this approach but also provides a foundation for further exploration of cross-cultural variations in normative influence.

One of the key contributions of our study is the experimental evidence regarding cross-cultural differences in response to social norm messages, including the shaping of normative perceptions. This novel insight extends the norm literature by shedding light on how culture can impact peoples’ responses to normative cues. By demonstrating how different cultural contexts may lead to distinct interpretations and salience of the same norms message, and how these internalized normative perceptions will further interact with one's cultural context to shape behavioural outcomes, we highlight the importance of considering cultural factors when designing norm-based interventions. These findings echo other literature, suggesting the critical role of norm perceptions as a *vehicle* in promoting collective actions for a large-scale social change [37], as interventions influence behavioural changes through a shift in perceived norms [38]. Our study provides new evidence that carefully designed persuasive messages can shape the norm perceptions in the desired direction and/or strengthen the perceived salience of an existing norm, such as food waste prevention, across different cultural contexts.

The findings also indicate that, counter to our predictions, group orientation did not interact with either the norms message or normative perceptions as expected. This is in contrast with previous literature, which suggests that a collectivistic orientation should enhance susceptibility to normative influence [46,55]. One explanation for this divergence is that we operationalized group orientation as an individual-level variable, in contrast to the country-level construct used in previous meta-analyses [46]. Our holistic analytical approach, which incorporated both cultural context (at the country level) and individual-level group orientation as potential moderators within the mediation model (message → perceptions → behavioural intentions), offers an opportunity to assess the individual and combined predictive influence of these moderators. Simultaneously examining both cultural and individual factors allowed us to gain nuanced insights into their respective effects on behavioural outcomes, while accounting for the interplay between these moderators.

Our study suggests that cultural context plays a more powerful role in shaping norm perceptions and subsequently impacting behaviours relative to the role of group orientation, indicating the need for further investigation in this domain. Notably, in a recent meta-analysis examining the effects of norm-based interventions on pro-environmental behaviours, including both descriptive and injunctive norms, Helferich *et al*. [11] found either no statistically significant evidence (using the GLOBE scale) or very weak but significant moderation effects (using Hofstede's scale) of individualism versus collectivism on both types of normative influence on intentions. The Helferich *et al*. [11] findings combined with the present study underscore the need to broaden the scope of sociocultural factors considered in norm-related research and intervention practices, moving beyond the individualistic versus collectivistic orientations, which has traditionally been central in intercultural and cross-cultural studies for several decades [50].

As previously reviewed, the emerging dimension, cultural tightness–looseness (CTL), may take centre stage in explaining our findings. It is possible that the prevailing cultural tightness in China, characterized by stringent norms and potential sanctions for deviations when contrasted with the more lenient atmosphere in the US [35], may contribute to heightened norm perceptions and subsequent behavioural intentions when the norm message exposure accentuates the focal norm. Remarkably, the cultural context also exerts a standalone main effect on both norm perceptions, parallel to the impacts of the message appeals, emphasizing the critical role of a society's cultural norms and overall cultural climate in promoting pro-social behaviours. Further research might consider the incorporation of elements such as CTL in tests of social norms-based messages.

### (a) Implications

The findings of this study, coupled with existing scholarship, provide several additional implications for theory advancement and practical utility in norm-based intervention programmes and campaign design within a cross-cultural context. By testing the potential cultural mechanisms of normative influence at both societal and individual levels, this study contributes to broadening the scope of the social norm literature and enhancing its explanatory and predictive power in diverse cultural settings. While the expected moderation effects of group orientation were not evident in our data, the cultural context was shown as an influential factor in predicting behavioural intentions through both standalone main effects and interactive effects with the norm appeals and perceptions.

Specifically, our findings suggest that an individual's cultural background has a more substantial influence on the normative effects in the context of food waste prevention, outweighing the influence of individual-level group orientation. Nevertheless, we did find that group orientation had a significant main effect on behavioural intentions, aligning with the collective nature of food waste reduction as a behaviour that benefits society as a whole. Future norm studies should explore the role of group orientation, especially at the individual level, across different issues and topics to replicate these findings.

Taken together, drawing insights from our data and the broader literature, it is evident that norm appeals do exert a substantial effect in shaping norm perceptions and promote subsequent behaviours transcending cultural boundaries. However, social norm appeals—either descriptive or injunctive norms—may be most effective when being implemented in cultural contexts where there has been an existing norm or evolving social environment aligned with the message exposure. The cultural backdrop magnifies the potency of norm appeals and norm perceptions in steering behaviours. This duality not only streamlines the process but also ensures a more impactful and efficient outcome, ultimately leading to amplified results with half the effort.

For cultural context characterized by looseness and/or in the absence of a strong existing norm encouraging people to engage in pro-environmental behaviours, message appeals may consider focusing on fostering or activating a stronger connection with referent groups, given the substantial main effect of group orientation in preventing food waste evident in our data. Alternatively, campaign designers may consider developing appeals incorporating dynamic norms. A growing body of literature [68,69] suggests the effects of presenting information emphasizing the increasing prevalence or social approval of certain behaviours. By drawing people's attention to the positive changes instead of the issue's current state, especially when it is undesirable, dynamic norm messages were shown to be more effective in motivating pro-environmental behaviours compared to static norm appeals [68].

In summary, by recognizing the importance of sociocultural factors, future norm-based intervention programmes and campaigns can be tailored to specific cultural contexts. Our research bridges the gap between theory and practice, providing valuable guidance for the design and implementation of culturally sensitive and contextually appropriate interventions that leverage normative influences effectively.

### (b) Limitations and future directions

Despite its merits, this study is not without limitations. One major limitation pertains to the external validity, or generalizability, of our results. The study's experimental design hinged on a fictional cover story, namely the food waste prevention program, *Waste Wise*, on campus. This approach, while essential for constructing the experimental message stimuli, may not wholly mirror real-world scenarios. The large size of the full student body (over 50 000 students at all universities where the data were collected) makes it unlikely, but possible, that participants would have doubted the cover story since they are members of the university community and might be aware of a previous food waste study conducted there.

In practical settings, the dynamics of social normative influence on behaviours could vary substantially, influenced by nuanced factors that are difficult to encapsulate within a controlled experimental environment. Consequently, there exists a potential gap between the observed effects within the confines of our experimental context and how these processes manifest in real-life situations. Additionally, despite incorporating measurements of message believability, this study did not include an assessment of demand characteristics; it remains unknown whether participants guessed the experimental aims and behaved in accordance with perceived researcher expectations. Future investigations may consider extending the experimental setting into real-world scenarios, such as conducting field experiments on a variety of pro-environmental topics, which may help elucidate the practical implications of our results in a more authentic context.

Another aspect warranting consideration is the sampling strategy and data collection modes employed in this study. The data were collected from samples of students from both countries, representing a specific demographic group; Chinese participants completed the survey in a face-to-face group setting, whereas American participants completed it individually online. The homogeneity of the sample could introduce limitations concerning the generalizability of our findings to a broader population. The unique characteristics, values and attitudes exhibited by students may not be representative of other age groups or demographics. Additionally, the differences in survey administration modes may have impacted the salience of social norms and contributed to the observed cultural variations in susceptibility to those norms. Therefore, while our findings offer valuable insights into the behaviours of students within the context of food waste prevention, extending these conclusions to diverse demographic groups necessitates further research involving a more representative sample.

Furthermore, it is essential to recognize that this study primarily focuses on behavioural intentions rather than actual behaviours. While intentions are strong predictors of subsequent actions, they do not always translate into real-world practices. Therefore, our findings should be interpreted with the understanding that they pertain to behavioural intentions rather than the tangible actions that individuals may undertake. Future research endeavours should seek to bridge this gap by incorporating measures of actual behaviours, providing a more comprehensive perspective on the impact of social normative influence cross-culturally.

Nonetheless, the findings of this study offer promising insights for future research endeavours. By delving deeper into the intricate relationship between culture, social norms and behaviours, we can advance our understanding of the underlying complexities involved. This will enable the development of more sophisticated and tailored social norm interventions that resonate with diverse cultural contexts and individual orientations, ultimately fostering greater success in promoting desired behaviours and social change.

## Data Availability

Supplementary material is available online [70].

## Endnotes

1 Because the study received approval from the IRB in the United States and shared all relevant documents with our Chinese collaborators and university administrators for their review before initiating data collection, the Ethics Review Board in the Chinese universities determined that no additional approval was necessary.

2 Yunnan is China's most ethnically diverse province, with 33% of the population from 25 official ethnic minorities (including 16 indigenous), representing nearly half of China's list of 55 officially recognized ethnic minorities (non-Han Chinese population).

3 This was necessary due to the absence of available digital platforms in China during the data collection period, which is a limitation of the study design.

4 All message stimuli have been pretested in a pilot study and are included in the electronic supplementary material (Appendix A).

5 See the detailed explanations of the procedures and the results in the electronic supplementary material (Appendix B).
